# Assessing the utility of Xpert^®^ MTB/RIF as a screening tool for patients admitted to medical wards in South Africa

**DOI:** 10.1038/srep19391

**Published:** 2016-01-20

**Authors:** Christine L. Heidebrecht, Laura J. Podewils, Alexander S. Pym, Ted Cohen, Thuli Mthiyane, Douglas Wilson

**Affiliations:** 1 Clinical and Biomedical Tuberculosis Research Unit, Medical Research Council, South Africa; 2 Division of Tuberculosis Elimination, Centers for Disease Control and Prevention, USA; 3 KwaZulu-Natal Research Institute for Tuberculosis and HIV (K-RITH), South Africa; 4 Division of Global Health Equity, Brigham and Women’s Hospital, USA; 5 Department of Epidemiology, Harvard School of Public Health, USA; 6 School of Laboratory Medicine and Medical Sciences, University of KwaZulu-Natal, South Africa; 7 Department of Medicine, Edendale Hospital, South Africa

## Abstract

Many hospital inpatients in South Africa have undiagnosed active and drug-resistant tuberculosis (TB). Early detection of TB is essential to inform immediate infection control actions to minimize transmission risk. We assessed the utility of Xpert^®^ MTB/RIF (GeneXpert) as a screening tool for medical admissions at a large public hospital in South Africa. Consecutive adult patients admitted to medical wards between March-June 2013 were enrolled; sputum specimens were collected and tested by GeneXpert, smear microscopy, and culture. Chest X-rays (CXRs) were conducted as standard care for all patients admitted. We evaluated the proportion of patients identified with TB disease through each diagnostic method. Among enrolled patients whose medical charts were available for review post-discharge, 61 (27%) were diagnosed with TB; 34 (56% of diagnosed TB cases) were GeneXpert positive. When patients in whom TB was identified by other means were excluded, GeneXpert yielded only four additional TB cases. However, GeneXpert identified rifampicin-resistant TB in one patient, who was initially diagnosed based on CXR. The utility of GeneXpert for TB screening was limited in an institution where CXR is conducted routinely and which serves a population in which TB and TB/HIV co-infection are highly prevalent, but it allowed for rapid detection of rifampicin resistance.

South Africa’s tuberculosis (TB) burden is among the highest in the world^1^, and it is the leading cause of death for South Africans between the ages of 15 and 64 2. Approximately 61% of TB cases are co-infected with HIV^1^ and in 2014 there were close to 19,000 cases of laboratory-confirmed multidrug-resistant (MDR) TB^1^. The burden of TB and MDR TB among medical inpatients in South Africa is particularly high^3^,^4^, and recent evidence suggests that many hospital patients have unsuspected TB^5^. This high prevalence, coupled with the open-ward design of South Africa’s public hospitals, creates conditions favouring nosocomial infection among inpatients and hospital staff^4^,^6^,^7^,^8^,^9^,^10^,^11^, representing an urgent need to improve screening of patients for TB and MDR TB upon admission.

Timely case identification and initiation of appropriate treatment are critical for TB control^12^,^13^ but diagnostic tools have been inadequate. Sputum smear microscopy is insensitive, particularly among HIV-infected individuals^14^, and cannot differentiate between drug-susceptible and drug-resistant strains of *Mycobacterium tuberculosis* (*MTB*). Sputum culture, the gold standard for detecting *MTB*, takes weeks to months to yield results, and relies on sophisticated laboratory facilities and skilled technicians.

Xpert® MTB/RIF (GeneXpert) is a newly-developed World Health Organization (WHO)-endorsed nucleic acid amplification test which detects both *MTB* and rifampicin (RIF)-resistance–a surrogate marker for MDR TB–and produces results in approximately two hours^15^. The cartridge-based system requires minimal training and biosafety measures, and can be implemented closer to the point of care than other tests^16^,^17^. Clinical validation trials^17^,^18^,^19^ and operational studies^20^,^21^,^22^,^23^ have demonstrated high sensitivity and specificity of the test for both *MTB* and RIF-resistance, but much work remains to ascertain the optimal application of GeneXpert in various settings and populations.

We conducted a study at a regional hospital in KwaZulu-Natal, South Africa to assess the value of GeneXpert as a TB screening tool for medical inpatients.

## Methods

### Setting and study population

This study was conducted at Edendale Hospital in Pietermaritzburg, South Africa, a 900-bed facility that serves a population living in peri-urban and rural settings. GeneXpert was implemented at the hospital in 2012 and has replaced smear microscopy as the primary tool for evaluating persons clinically suspected of having TB. We aimed to screen all adults admitted to Edendale’s five medical wards between March and June 2013 for TB disease using GeneXpert. Patients were eligible for enrollment if they had received fewer than three doses of TB treatment, could provide informed consent and could produce a respiratory specimen, regardless of TB signs or symptoms. New patients were identified through daily review of hospital admission registers and medical ward files. For logistical reasons of staff availability to collect sputum samples immediately upon admission (i.e., before any treatment was initiated), only patients who were admitted between Sunday and Friday morning were included.

### Sample and data collection

Each consenting patient was asked to provide two sputum samples. The first sample was collected upon enrollment and the second was collected later that day or the following day (both within 24 hours of admission). Sputum was not induced unless requested by the physician. The first sputum sample was tested with GeneXpert on-site at Edendale’s laboratory by a study technician, and the second was transported to a research laboratory for microscopy, culture, and drug susceptibility testing (DST). Regardless of volume or consistency, all specimens were tested.

GeneXpert results were placed in each participant’s ward file. Culture results positive for *MTB* were shared with the hospital’s TB team.

Patient data were obtained through questionnaires and chart abstraction. Study personnel reviewed admission files, ward files and patient charts to collect demographic and clinical data. Prior to the availability of laboratory results, patients were interviewed by research staff to verify eligibility (i.e. age, less than three doses of TB treatment), document previous TB history, and obtain information on TB signs and symptoms. Persons with presumptive TB were defined as patients whose admitting diagnosis suggested possible TB (based on medical chart abstraction) or for whom a TB test was ordered at admission. A patient was defined as being diagnosed with TB disease if TB was documented as the final diagnosis or TB treatment had been initiated by the time of discharge. Chest radiography (CXR) is routinely performed on all patients upon admission. File notes describing CXR were classified by clinical research staff as indicative of TB, “unknown”, or not indicative of TB. For analysis, films classified as indicative of TB or “unknown” were grouped together as “abnormal.”

### Laboratory procedures

GeneXpert testing was performed on raw specimens in accordance with manufacturer’s instructions^24^.

Samples collected for smear microscopy and culture were transported to a Medical Research Council laboratory and stored between 2–8 °C until processed. Smears were prepared using auramine- and Ziehl Neelsen-stained slides. Both MGIT and 7H11 Middlebrook were used for culture; DST for first-line drugs was performed on culture-positive samples.

### Statistical analysis

Data were double-entered into an Epi Info database, and all analyses were conducted in Stata 13 (College Station, TX, USA). Comparisons between eligible and ineligible patients, and between participants in whom TB was detected or diagnosed and those in whom it was not, were made using Pearson’s chi-square test or Fisher’s exact test. The Wilcoxon rank-sum test was used to compare median time-to-treatment between GeneXpert-positive and -negative patients.

### Ethics approval

This study was approved by the research ethics committees of the South African Medical Research Council, the province of KwaZulu-Natal’s Department of Health, Partners HealthCare and the US Centers for Disease Control and Prevention. Permission to conduct the study was granted by Edendale Hospital. All study participants provided written informed consent prior to initiation of study activities.

## Results

### Patient characteristics

**Figure 1:**
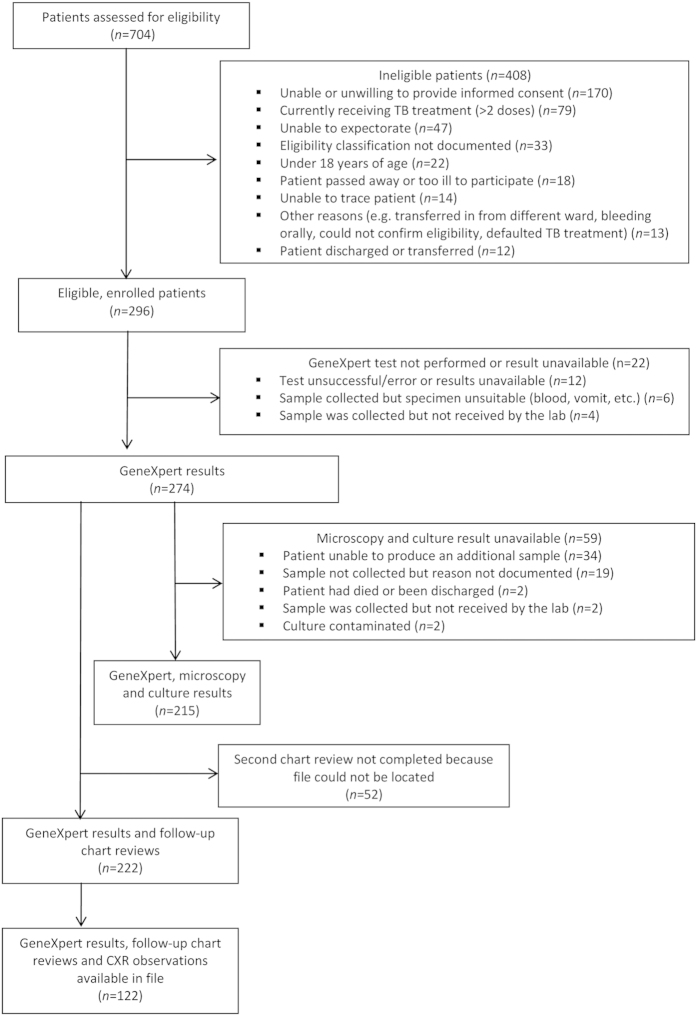
Eligibility of patients admitted to medical wards at Edendale Hospital, KwaZulu-Natal, South Africa March–June 2013.

Of 704 patients admitted to medical wards during the recruitment period, 296 (42.0%) were eligible and enrolled (Fig. 1). The majority of patients were excluded because they were unable or unwilling to provide informed consent (170, 41.7%), had received more than two doses of anti-TB therapy (79, 19.4%), or could not expectorate (47, 11.5%). Enrolled patients did not differ significantly from ineligible patients with respect to sex (p = 0.48); participants tended to be younger than non-participants but this difference was not significant (p = 0.45). Close to two-thirds (64.9%) of enrolled patients were female, the median age was 41 years (IQR: 31–57), and the majority (61.7%) of participants with known status were HIV positive (Table 1).

Twelve (4.1%) of the 296 enrolled study participants had received one or two doses of TB treatment prior to enrollment. Among the 284 patients who had not initiated TB therapy at enrollment, 129 (45.4%) patients were considered persons with presumptive TB upon admission. Other respiratory illnesses (20.3%), cardiovascular conditions (17.9%), and TB (15.8%) comprised the most common admitting diagnoses.

### GeneXpert results and performance characteristics

GeneXpert results were available for 274 (92.6%) enrolled patients. Forty-four participants (16.1%) were GeneXpert positive for TB, and RIF-resistance was detected in four individuals (9.1%). Unadjusted comparisons between GeneXpert-positive and -negative patients indicate that the proportion of patients who were GeneXpert-positive was greater among patients who were younger, infected with HIV, had CD4 counts <350, or who had current or recent experience of three or more TB symptoms (Table 2).

Among patients for whom post-discharge file reviews were available (n = 222), 34 were GeneXpert positive, and of these, 28 were considered persons with presumptive TB at admission. GeneXpert therefore yielded six (17.6%) cases that were not suspected on admission; this yield was reduced to four (11.8%) when patients whose CXR indicated TB were excluded. RIF-resistance was not detected in these four patients but was detected in one of the two whose CXR indicated TB.

GeneXpert performance was assessed against smear and culture results from specimens of equivalent quality. For this comparison, 126 patients had both smear and GeneXpert results available and 125 patients had both culture and GeneXpert results available. Smear microscopy was positive in less than half (14/29, 48.3%) of GeneXpert-positive patients. The overall sensitivity of GeneXpert compared to culture was 82.1% (95% CI: 63.1, 93.9) and specificity was 93.8% (95% CI: 87.0, 97.7). Of the four cases of RIF resistance detected by GeneXpert, one was found to be drug susceptible after culture, drug susceptibility results were not available for two, and MTB was not isolated by culture in the fourth.

### Hospital diagnoses at discharge

**Figure 2:**
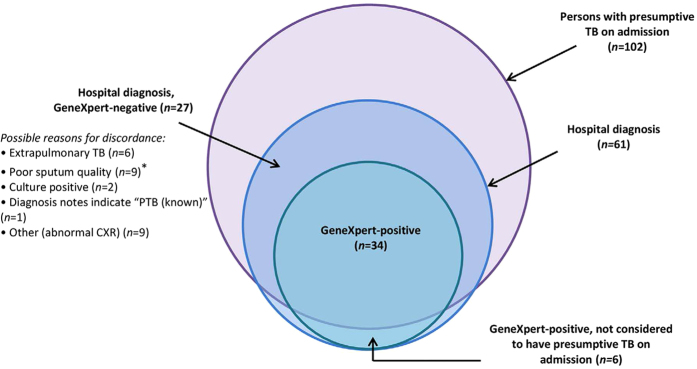
Hospital diagnoses and GeneXpert results for patients enrolled in the current study with post-discharge chart reviews (*n* = 222). *Saliva and/or low volume, or food particles.

Post-discharge file reviews were conducted for 222 (81.0%) enrolled patients for whom GeneXpert results were available, of whom 61 (27%) were diagnosed with TB. GeneXpert-positive patients comprised 55.7% (*n* = 34) of all diagnosed cases; all patients with a positive GeneXpert result were diagnosed with TB (Fig. 2). In addition to bacteriological tests, TB diagnoses were informed by CXR, extra-pulmonary imaging and/or clinical observations. Compared to GeneXpert, CXR had poor specificity (27.8%; 95% CI: 19.2, 37.9) but relatively high sensitivity (96.0%; 95% CI: 79.6, 99.9); among patients for whom GeneXpert and CXR results were available (*n* = 122), all but one GeneXpert-positive case had an abnormal CXR. Among those who were not considered persons with presumptive TB at admission, all GeneXpert-positive cases had abnormal CXRs.

Among patients who were diagnosed with pulmonary TB, there was no significant difference in the median time between admission and treatment initiation for GeneXpert-positive patients (2 days; IQR: 1,3) and those who were GeneXpert-negative and diagnosed though other modalities (1 day; IQR: 0,5) (*p* = 0.41).

## Discussion

Since the WHO’s endorsement of the assay in 2010, operational research studies have been conducted to examine GeneXpert’s utility in a range of settings and populations. In this study, we assessed the utility of GeneXpert as a screening tool for use among newly-admitted medical inpatients.

All consenting adults who could expectorate were screened for TB, regardless of signs and symptoms. Among patients who were not classified as having presumptive TB at admission and whose CXR findings did not indicate TB, four were GeneXpert-positive; symptoms reported by two of these patients suggest that they may have been investigated for TB in the absence of this intervention. Because GeneXpert results were provided to clinicians regardless of test orders, we do not know which investigatory actions may have been taken outside of the context of this study. However, we presume that patients who had a test ordered at admission, an admitting diagnosis of TB, and/or an abnormal CXR would have been investigated for TB. It is therefore likely that our intervention yielded few individuals whose TB would have gone undetected. Where skills and resources permit, screening for active TB clinically (symptoms, examination and imaging) may be the most effective strategy, reserving GeneXpert for confirmatory testing.

Notwithstanding these observations, inpatient screening is a comprehensive approach to case detection, and screening with GeneXpert may have expedited clinicians’ decision-making for persons with RIF-resistance. Further, targeted GeneXpert screening may yield a greater number of additional, unsuspected TB cases in settings with less access to CXR and/or fewer personnel skilled in clinical evaluation. Despite concessional pricing in high-burden and developing countries^25^, GeneXpert remains costly, however; it may be cost-effective to incorporate a triage or adjunct test^26^,^27^ into screening algorithms. Had GeneXpert screening been limited to persons with presumptive TB and participants with abnormal CXR findings, 20 (16%) GeneXpert tests could have been avoided without overlooking any GeneXpert positive patients. In a community-based study assessing persons with presumptive TB, Theron *et al.*^26^ found that CXR combined with GeneXpert (in patients whose CXR was suggestive of TB) did not differ with respect to predictive value when compared to GeneXpert alone and was thus less cost-effective. However, in a setting such as Edendale where all inpatients undergo CXR, the outcome may differ. van Hoog *et al.*^27^ describe the potential cost-effectiveness of a hypothetical triage test to complement GeneXpert; while CXR in the current setting does not meet the specificity and cost parameters of the test hypothesized, it may offer cost savings in a setting where CXR is routine.

We observed that a large proportion of inpatients who were diagnosed with TB while hospitalized were GeneXpert-negative. While some of these patients may have received false-negative results due to poor sample quality, this finding suggests a practice of presumptive treatment: in the presence of symptoms and other clinical indications (e.g., CXR consistent with TB), clinicians may treat patients empirically without bacteriological confirmation^28^. Recent observational studies conducted in institutional and community settings have demonstrated that empirical treatment is reduced in the presence of GeneXpert when compared to smear microscopy^29^,^30^,^31^,^32^. While our study design did not allow us to compare rates of empirical treatment before and after the introduction of GeneXpert, our results are consistent with two of these studies suggesting that empirical treatment occurs regardless of GeneXpert availability^29^,^30^, and support Theron *et al.*’s concern that the introduction of GeneXpert as a diagnostic tool might primarily reduce true-positive empirical treatment and consequently have less impact than anticipated on case identification^28^. Empirical treatment is unavoidable in settings where smear-microscopy is the only first-line diagnostic tool available, especially in populations with high HIV prevalence, but the extent to which it is appropriate in the context of GeneXpert availability warrants discussion.

The value of GeneXpert as a screening tool likely also depends on the epidemiology of a facility’s catchment population. In populations where TB is prevalent, screening approaches of relatively low specificity (CXR, symptom screening, etc.) are more likely to correctly identify cases than in settings where TB is rare. Similarly, presumptive TB diagnoses may be more accurate in persons co-infected with HIV due to the greater likelihood of HIV-positive individuals to present with TB. Further, though more sensitive than smear microscopy in this population, GeneXpert is less sensitive among individuals with HIV than in HIV-negative patients. This study was conducted in a facility that treats a patient population in which both TB and TB/HIV co-infection are highly prevalent; GeneXpert may be of greater benefit in settings where rates of either or both diseases are lower.

Case identification, as examined in this study, is only one parameter on which to base a conclusion about the utility of a diagnostic test. Studies comparing morbidity and/or mortality of patients tested with GeneXpert with other approaches have not observed significant differences in these outcomes^30^,^31^,^32^, which may be due in to part to clinicians’ propensity to empirically treat in some settings^30^. These studies could not or did not specifically look at morbidity or mortality among patients with drug-resistant TB, where GeneXpert may offer greater impact on clinical outcomes. In addition, Churchyard *et al.* recognize that GeneXpert testing may have improved detection and time to appropriate treatment for drug-resistant cases, thereby potentially reducing transmission of drug-resistant disease^32^. This is consistent with our anticipation when developing the present study that GeneXpert would rapidly identify drug resistant TB cases and prompt a meaningful infection control response, an impact of particular importance in clinical environments or communities with a high proportion of immunocompromised individuals.

This study has important limitations. A large proportion of inpatients were excluded due to their inability to provide informed consent; therefore, our study population may not be representative of all medical inpatients at Edendale. Additionally, the information abstracted from patients’ medical records was not documented for research purposes. The quality of data on which we based patient classifications of persons with presumptive TB or hospital-diagnosed TB cases was dependent on the comprehensiveness of clinicians’ record-keeping and study personnel interpretation of notes; we may have underestimated the true number of patients that would have been investigated for TB through routine procedures. Further, we were unable to collect a second sputum sample and/or conduct post-discharge file reviews for a large number of participants. The sub-samples of patients available for inclusion in the assessment of GeneXpert performance and comparisons between GeneXpert-positivity and hospital diagnosis were smaller than our original sample, and impacted our ability to draw meaningful conclusions. However, the observed sensitivity and specificity of GeneXpert were similar to measures reported in previous hospital-based studies using culture as the reference standard^20^,^22^,^31^. The discrepancy between GeneXpert-based and culture-based detection of RIF-resistance highlights the difficulties in confirming MDR TB in resource-limited contexts. Our estimate of cases whose TB would likely have been undetected under routine practices was restricted to patients with files available for post-discharge audits. It is possible that had we been able to review post-discharge files for all GeneXpert-positive patients, we may have observed a different yield attributable to GeneXpert.

## Conclusions

While its yield is greater than smear microscopy, the utility of GeneXpert as a TB screening tool may be limited in settings where CXR and initiation of TB therapy without microbiological confirmation is routine, and where TB and TB/HIV co-infection are highly prevalent. Regardless of available resources, the ability of GeneXpert to rapidly identify RIF-resistance and inform infection control procedures strengthens the rationale for its use. Rapid detection of both drug-resistant and -susceptible strains of TB, followed by appropriate treatment initiation, and isolation or transfer where applicable, are especially critical in inpatient settings to reduce mortality and risk of nosocomial transmission among immunocompromised patients. Depending on available resources, GeneXpert should be considered as either a confirmatory aid or primary screening tool in institutions serving TB-prevalent populations.

## Additional Information

**How to cite this article**: Heidebrecht, C. L. *et al.* Assessing the utility of Xpert® MTB/RIF as a screening tool for patients admitted to medical wards in South Africa. *Sci. Rep.* **6**, 19391; doi: 10.1038/srep19391 (2016).

## Figures and Tables

**Table 1:** Characteristics of patients enrolled in the present study evaluating the utility of GeneXpert for diagnosing TB, Edendale Hospital, KwaZulu-Natal South Africa March-June 2013 (*n* = 296).

Characteristic	*n*(%)
Sex	
Female	192 (64.9)
Male	103 (34.8)
Unknown	1 (0.3)
Age (years)	
18–29	68 (23.0)
30–44	93 (31.4)
45–59	75 (25.3)
60–74	44 (14.9)
75+	16 (5.4)
HIV status*	
Positive	161 (54.4)
Negative	100 (33.8)
Unknown/not recorded	35 (11.8)
Low CD4 count (<350 cells/ml)**	71 (71.7)
*TB risk factors and symptoms*†	
Previous TB	68 (23.4)
Current or recent cough	198 (67.1)
Current or recent weight loss	186 (63.1)
Current or recent fever	162 (54.9)
Current or recent loss of appetite	154 (52.7)
Current or recent night sweats	132 (45.2)
Total symptoms	
0–2 symptoms	121 (40.9)
3–5 symptoms	168 (56.8)
Not recorded	7 (2.4)
Admitted to hospital in past year	67 (23.0)
Ever served time in a prison	18 (6.3)
Ever worked in a mine	8 (2.8)
Ever lived in a household with a TB patient	74 (25.7)

^*^Based on self-reported data and hospital records.

^**^Among HIV+ patients for whom a CD4 count was available: n = 99 (61.5%).

^†^Unless otherwise noted, proportions exclude missing values; missing values comprised <5% of total for all variables.

**Table 2:** Demographic and clinical characteristics of patients by GeneXpert status (*n* = 274)*.

	GeneXpert positive (*n* = 44) *n*(%)	GeneXpert negative (*n *= 230)*n*(%)	*p*-value**
Sex			
Female	28 (63.6)	146 (64.8)	0.86
Male	16 (36.4)	80 (34.8)	
Unknown	0	1 (0.4)	
Age			
18–29	16 (36.4)	49 (21.3)	<0.01
30–44	17 (38.6)	70 (30.4)	
45–59	10 (22.7)	55 (23.9)	
60–74	1 (2.3)	40 (17.4)	
75+	0	16 (7.0)	
HIV status			
Positive	35 (79.5)	111 (48.3)	<0.01
Negative	7 (15.9)	88 (38.3)	
Unknown/not recorded	2 (4.5)	31 (13.5)	
Low CD4 count (<350 cells/mL)†	18 (94.7)	48 (65.8)	0.01
*TB risk factors and symptoms*††			
Previous TB	13 (29.6)	47 (20.9)	0.21
Current or recent cough	37 (86.1)	142 (61.7)	<0.01
Current or recent weight loss	38 (86.4)	134 (58.5)	<0.01
Current or recent fever	31 (70.5)	118 (51.5)	0.02
Current or recent loss of appetite	32 (74.4)	107 (47.1)	<0.01
Current or recent night sweats	26 (59.1)	96 (42.5)	0.04
Total symptoms			
0–2 symptoms	10 (22.7)	105 (45.7)	<0.01
3–5 symptoms	32 (72.7)	120 (52.2)	
Not recorded	2 (4.6)	5 (2.2)	
Admitted to hospital in past year	5 (11.4)	58 (25.7)	0.05
Ever served time in a prison	2 (4.8)	15 (6.7)	1.00
Ever worked in a mine	0	8 (3.6)	0.36
Ever lived in a household with a TB patient	12 (27.3)	56 (25.1)	0.76

^*^Study personnel reviewed admission files, ward files and patient charts to collect demographic and clinical data. Prior to the availability of laboratory results, research staff administered a participant questionnaire containing items concerning TB history, symptoms, possible TB exposure, and risk factors.

^**^Pearson’s chi-square (excludes unknown values); Fisher’s exact test used when one or more cells contained a value of ≤5.

^†^Among HIV+ patients for whom a CD4 count was available (92, 63%); *n*(GeneXpert positive) *= *73 *n*(GeneXpert negative) = 19.

^††^Unless otherwise noted, proportions exclude missing values; missing values comprised <5% of total for all variables.
